# Multivariate Statistical Models for the Authentication of Traditional Balsamic Vinegar of Modena and Balsamic Vinegar of Modena on ^1^H-NMR Data: Comparison of Targeted and Untargeted Approaches

**DOI:** 10.3390/foods12071467

**Published:** 2023-03-29

**Authors:** Eleonora Truzzi, Lucia Marchetti, Danny Vincenzo Piazza, Davide Bertelli

**Affiliations:** Department of Life Sciences, University of Modena and Reggio Emilia, Via Campi 103, 41125 Modena, Italydavide.bertelli@unimore.it (D.B.)

**Keywords:** qNMR, linear discriminant analysis (LDA), chemometrics, Traditional Balsamic Vinegar of Modena, Balsamic Vinegar of Modena, principal component analysis (PCA)

## Abstract

This work aimed to compare targeted and untargeted approaches based on NMR data for the construction of classification models for Traditional Balsamic Vinegar of Modena (TBVM) and Balsamic Vinegar of Modena (BVM). Their complexity in terms of composition makes the authentication of these products difficult, which requires the employment of several time-consuming analytical methods. Here, ^1^H-NMR spectroscopy was selected as the analytical method for the analysis of TVBM and BVM due to its rapidity and efficacy in food authentication. ^1^H-NMR spectra of old (>12 years) and extra-old (>25 years) TVBM and BVM (>60 days) and aged (>3 years) BVM were acquired, and targeted and untargeted approaches were used for building unsupervised and supervised multivariate statistical modes. Targeted and untargeted approaches were based on quantitative results of peculiar compounds present in vinegar obtained through qNMR, and all spectral variables, respectively. Several classification models were employed, and linear discriminant analysis (LDA) demonstrated sensitivity and specificity percentages higher than 85% for both approaches. The most important discriminating variables were glucose, fructose, and 5-hydroxymethylfurfural. The untargeted approach proved to be the most promising strategy for the construction of LDA models of authentication for TVBM and BVM due to its easier applicability, rapidity, and slightly higher predictive performance. The proposed method for authenticating TBVM and BVM could be employed by Italian producers for safeguarding their valuable products.

## 1. Introduction

The Italian province of Modena is celebrated in the world for its numerous typical and well-appreciated food products that obtained the protected designation of origin (PDO) or protected geographical indication (PGI). Certainly, the Traditional Balsamic Vinegar of Modena (TBVM) and the Balsamic Vinegar of Modena (BVM) are among the most famous Italian foods protected by geographical indications for their high historical and economical values.

Faithfully to the ancient tradition and in accordance with the actual regulations (EC Council Regulation No. 813/2000), TBVM is obtained only from the alcoholic and acetic fermentation of cooked must from grapes harvested in Modena with the denomination of controlled origin. Besides, the production of the less expensive BVM also allows the use of concentrated grape musts, wine vinegar (10% *v*/*v* minimum), and flavoring such as caramel (2% *v*/*v* maximum) (Reg. CE No. 583/2009 3 July 2009). Another fundamental difference between these two kinds of vinegar is the production procedure. The aging of TBVM requires transferring the starting material (cooked must) into a series of wooden casks of decreasing volume to obtain the desired high-density product. Sets of wooden casks can be made of different woods and are usually composed of five barrels. Every year, a portion of the final product (old TBVM) is withdrawn from the smallest barrel (cask No. 1), which is refilled with a variable volume from the previous barrel (cask No. 2). This process is performed up to the biggest barrel (cask No. 5) that is filled up with freshly cooked must. This process demands at least 12 or 25 years to obtain an old or an extra-old TBVM, respectively [1]. In the case of BVM, the aging period is strongly shorter (at least 60 days) and involves the maturation of vinegar in bigger barrels. The BVM is defined as “aged” when the aging lasts for over 3 years in barrels. Considering the above-mentioned procedures, the final result of the products is considerably different. TBVM is valuable, expensive, and appreciated by demanding consumers, whereas the cheaper BVM is suitable for being easily marketed to large distribution networks.

The composition of these products is very complex and not completely elucidated, especially considering the minor constituents and the high molecular weight compounds derived from caramelization and Maillard reactions [1,2]. In addition, it should be considered that TBVM is often produced in small companies, following traditional habits, and familiar recipes passed down for centuries. Consequently, the final product is susceptible to a very high and natural composition variability. This kind of variability is less relevant for BVM, which is instead made by more industrialized processes. To assure the quality and authentication of the different kinds of balsamic vinegar and to preserve their high economical value and reputation, numerous chemical–physical parameters have to be measured. The main constituents of the two kinds of vinegar are sugars (glucose and fructose in particular), sugar derivatives, organic acids, 5-hydroxymethylfurfural, and other compounds synthesized during fermentation [3,4,5,6,7]. In the literature, several authors focused on the chemical characterization of TBVM and BVM by employing chromatographic analytical methods to protect these products from counterfeiting [5,8,9,10,11,12,13,14]. However, these separative methods are time-consuming and require the employment of a high number of experiments to obtain the overall characterization of products.

Over recent years, the use of NMR and quantitative NMR (qNMR) spectroscopy for the analysis and characterization of complex matrices has flourished, demonstrating many advantages with respect to separative techniques [15,16,17,18]. Indeed, NMR spectroscopy does not require any complex sample preparation and the acquisition of a protonic spectrum takes just a few minutes. Additionally, the intensity of the spectral signal is influenced by the number of nuclei in the sample. Thus, the quantification of the target compounds is achieved in one analysis only through the employment of a unique universal standard (internal or external).

NMR spectroscopy can be applied in metabolomics to quantify and recognize compounds in complex biological or phytochemical samples. In recent years, it was extensively used for the characterization of valuable food products, such as wines, edible oils, honey, and vinegar, among others [16,19,20]. Additionally, NMR spectroscopy demonstrated great potential and advantages for the authentication of valuable foods [21]. Two different approaches can be used for authenticating foods, the targeted and the untargeted ones. The targeted approach is based on the quantification of target molecules, which are well recognized as important metabolites for the authentication of a product. The untargeted strategy is based on the determination of a high number of chemical parameters without any preliminary selection of the most important ones [18]. For this strategy, NMR spectroscopy is one of the most important analytical tools providing a fingerprinting profile of foods. To date, NMR spectroscopy has been employed a few times on TBVM and BVM [6,11,22,23,24,25,26]. In the present work, we aimed at the employment of both targeted and untargeted approaches coupled with multivariate statistical analysis for building preliminary authentication models. For the first time, the different strategies were compared to identify the most suitable method for rapid quality control of TBVM and BVM. 

## 2. Materials and Methods

### 2.1. Materials

A total of 57 samples of both TBVM and BVM have been analyzed. The 36 samples of TBVM were of different ages from 12 to over 25 years (14 old and 22 extra-old). Among the 21 samples of BVM, 13 were defined as aged. All kinds of vinegar samples were provided by private producers. Each sample belonged to a different set of barrels. Pyridoxine, dimethyl sulphoxide-*d6* (DMSO-*d6*), and 3-(Trimethylsilyl)propionic-2,2,3,3-d4 acid sodium salt (TSP) for internal referencing were purchased from Sigma-Aldrich (Milan, Italy). Citric, malic, succinic, lactic, acetic, formic, and tartaric acids, ethyl acetate, glucose, fructose, 6-*O*-acetyl glucose, acetyl fructose, 5-hydroxymethylfurfural (5-HMF), leucine, valine, 2,3-butanediol, and ethanol were provided by Sigma-Aldrich (Milan, Italy).

### 2.2. Sample Preparation and Spectra Acquisition Procedure

Fifty μL of each sample was weighted and diluted into the Wilmad^®^ NMR tube (5 mm, Ultra-Imperial grade, 7 in. L, 526-PP, Sigma-Aldrich, Milan, Italy) with 550 μL of DMSO-*d6*. Pyridoxine standard solution (4.98 mM) in DMSO-*d6* was selected as an external reference compound for quantification. Reference standards of organic acids, sugars, and compounds generated from the fermentation process were solubilized in acidified water at the same pH of vinegar, and 50 μL of the solutions were diluted with 550 μL of DMSO-*d6*. 

Spectra were acquired with a Bruker FT-NMR Avance III HD 600 MHz spectrometer (Bruker Biospin GmbH Rheinstetten, Karlsruhe, Germany) equipped with a CryoProbe BBO H&F 5 mm. All the experiments were performed at 300 K and non-spinning.

After the sample introduction into the probe, at least 5 min must be waited to achieve the thermal equilibrium. Afterward, the magnetic field was locked, the probe head was tuned and matched, and the sample was shimmed. To assure the highest reproducibility, all these procedures were automatically executed. For each sample, the correct 90° pulse was calibrated with the Bruker AU program “pulsecal”, and the receiver gain was set.

^1^H-NMR data were acquired using the Bruker sequence “zgcppr” for residual water presaturation. Acquisition parameters for “zgcppr” were as follows: time domain (number of data points), 64 K; dummy scans, 2; acquisition time, 3.90 s; delay time, 5 s; pulse width, 12 μs; number of scans, 64; spectral width, 14 ppm (8403.4 Hz); fid resolution, 0.1282 Hz; digitalization mode, baseopt. The total acquisition time was 6 min and 49 s. Since the use of the correct delay time (D1) is fundamental to assure the accurate quantification of considered compounds, the exact T1 for all the analytes and pyridoxine protons were measured using the Bruker Sequence “T1IR”, and the acquisition parameters were as follows: a list of 10 increasing delay times (from 10 ms to 30 s); delay time, 30 s; number of scans, 1; total acquisition time, 6 min and 54 s. A D1 time equal to five times the biggest T1 was used [27].

The acquired spectra were baseline corrected, phased, and referenced to TSP on Mnova^®^ 14.1.2 software (Mestrelab Research, S.L., Santiago de Compostela, Spain). The processed spectra were aligned and cut to remove the solvent peak and regions without signals and exported as spectral intensities to generate the untargeted dataset for the following statistical analysis. 

### 2.3. qNMR 

The quantification of target compounds was carried out using the Concentration Conversion Factor (CCF) method, implemented in Mnova^®^. The Mnova tool requires a multiplet analysis for the integration. Therefore, after the initial spectra processing, a manual multiplet analysis was carried out, and the peak area of signals belonging to the target compounds was compared to the area of signals generated by the pyridoxine standard solution (external reference) [27]. The pyridoxine solution in DMSO-*d_6_* (4.98 mM) was prepared immediately before the acquisition and analyzed under the same experimental parameters. ^1^H-NMR signals of pyridoxine used for the quantification were singlet at 7.83 ppm (corresponding to the aromatic proton in C6), singlet at 4.59 ppm (corresponding to two protons in C5′), and singlet at 2.40 ppm (corresponding to the three protons in C2′).

For the quantification purpose only resonances with a sufficient signal-to-noise ratio (at least 100:1), during the spectra transformation an exponential window function, with a line broadening (lb) equal to 0.3, were applied. 

In order to confirm the efficacy of this method, several spiked samples of TBVM (*n* = 3) and BVM (*n* = 3) were created by adding a solution containing various analytes at concentrations ranging from 0.1 to 10 g/L, taking into account their natural concentration in samples. The results are reported as mean recovery ratio. The precision was evaluated by preparing and analyzing ten times the same TBVM sample and comparing areas from the resonances used for quantification. The precision is expressed as averages of single CV% and results of 1.39%.

### 2.4. Determination of Solid Soluble Content

The solid soluble content (SSC) of vinegar samples was determined through an ABBE refractometer (Atago Co., Milan, Italy) by depositing one drop of vinegar onto a flint glass prism and measuring the degree Brix (°Bx) [28]. The analysis was performed in triplicate for each sample. 

### 2.5. Statistical Analyses

#### 2.5.1. Univariate Statistical Analysis

Data are expressed as mean ± standard deviation (SD). A Shapiro–Wilk normality test was carried out to assure that all data were normally distributed. Then, a one-way analysis of variance (ANOVA), followed by Tukey’s post hoc test, were performed on the quantitative results of the qNMR method. 

#### 2.5.2. Multivariate Statistical Analyses

Prior to the multivariate analyses, quantitative data were preprocessed by autoscaling. Moreso, spectral data were pretreated by means of baseline (Automatic Whittaker Filter, asymmetry = 0.001, lambda = 100) to reduce the spectral noise, followed by Pareto scaling and mean-centering. Pareto-scaling is the preferred treatment for NMR data for adjusting the magnitude of each variable without increasing the noise [17]. 

Principal component analysis (PCA) was performed on PLS_Toolbox for MATLAB^®^ (version 8.9.2, Mathworks Inc., Natick, MA, USA) on the targeted and untargeted datasets. The bidimensional matrices were composed of 58 samples × 17 or 37,656 variables for the targeted and untargeted datasets, respectively. The cross-validation for all the statistical models was performed by using the Venetian-blind method with 10 data splits. The number of principal components (PCs) was selected according to the smallest root mean squared error in calibration (RMSEC) and cross-validation (RMSECV) [17]. 

Linear discriminant analysis (LDA) was performed on IBM SPSS statistics (version 26, Armonk, New York, NY, USA). The leave-one-out cross validation method was selected for both models. The targeted dataset was employed as it was for the classification LDA model, whereas the untargeted dataset was reduced by eliminating those variables with a factorial weight lower than 0.85 in factorial analysis (less influential variables) [24]. In the latter case, a stepwise analysis was also carried out by employing Wilks’ lambda method for further variable selection. Variable reduction and stepwise analysis were necessary since the number of variables in the untargeted dataset highly exceeded the number of samples.

## 3. Results and Discussion

Each complex mixture, such as vinegar, is characterized by a specific and unique chemical fingerprint. Fingerprint recognition can be achieved by several complementary analytical techniques through two different strategies: the untargeted and the targeted. In our previous work, the recognition and discrimination of old and extra-old TBVM were successfully achieved using the NMR fingerprinting untargeted approach [24]. In the present work, the targeted approach based on qNMR analysis was compared to the NMR fingerprinting untargeted approach for the discrimination of high- and low-quality BVMs and old and extra-old TBVM. 

In a previously published work, Caligiani et al. tested the qNMR on vinegar using a manual integration procedure on samples dissolved in water [29]. In the present work, an automated procedure of peak integration and deconvolution was used.

**Figure 1:**
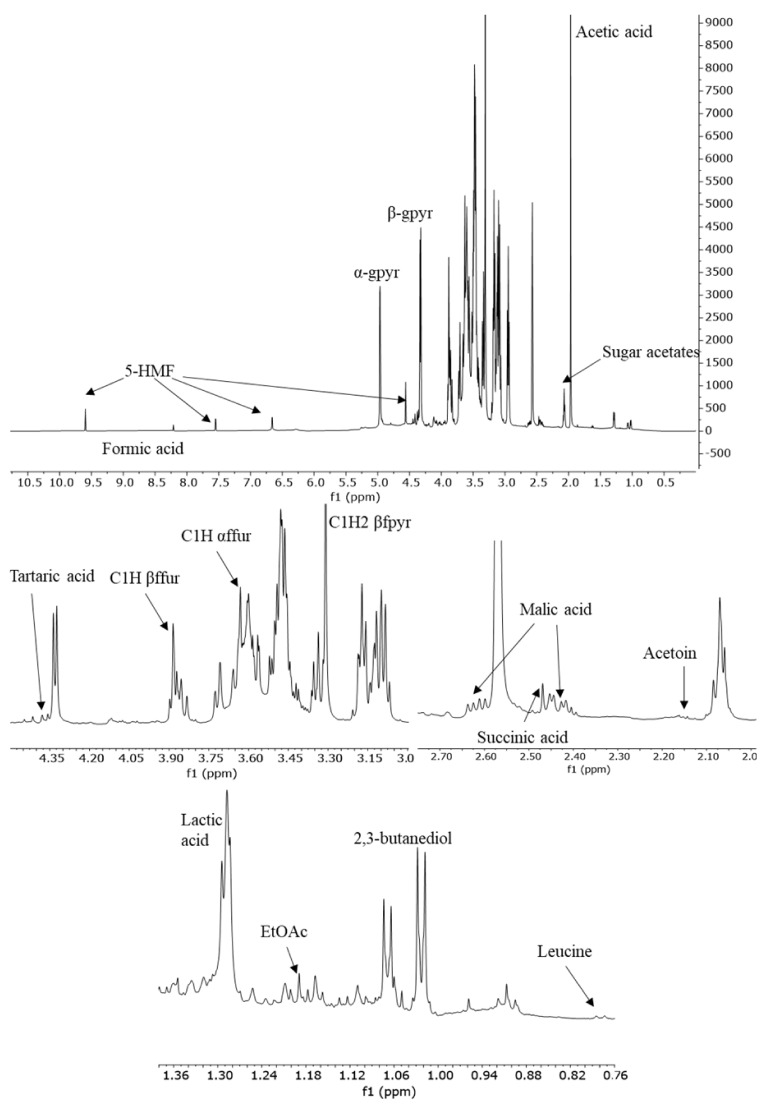
^1^H-NMR spectrum of a Traditional Balsamic Binegar of Modena. EtOAc, ethyl acetate; ffur, fructofuranose; fpyr, fructopyranose; gpyr, glucopyranose; 5-HMF, 5-hydroxymethylfurfural.

Figure 1 shows a typical ^1^H-NMR spectrum recorded at 600 MHz of a TBVM sample in DMSO-d_6_. The assignments of the most important metabolites were reported in previously published work [24,26]. In Table 1 signals used for the quantification of different compounds are listed. 

First, the T1 relaxation time of each proton of principal metabolites was determined. The longest T1 relaxation times (over 1.5 s) were achieved by C1H (s, 9.60 ppm), C3H (d, 7.55 ppm), and C4H (d, 6.66 ppm) of 5-HMF. All the other protons showed T1 values equal to or below 1 s. Considering that long relaxation times would convert the ^1^H-NMR spectroscopy into a time-consuming analysis, a delay time of 5 s was chosen. Consequently, the above-listed signals of 5-HMF were not selected for quantification. 

For the determination of 2,3-butanediol and acetoin, two signals were used, and the contents were reported as the average.

Concerning sugars, glucose, fructose, and their acetates were identified in TBVM and BVM proton NMR spectra. For the quantification of glucose and fructose, the final concentration was calculated by summing the abundances of the detected tautomeric forms. Specifically, among tautomers in the aqueous solution of fructose, only α- and β-furanose and the β-pyranose conformations could be identified in the NMR spectra, according to the literature reports [30]. Concerning glucose, α- and β-pyranose tautomers represent 99% of the carbohydrate in solution [31]. On the contrary, the tautomeric forms of glucose and fructose acetates could not be discernable due to their complexity and lack of NMR data in the literature. The whole group of signals in the range from 2.04 to 2.10 ppm related to methyl groups of acetates was integrated and the quantification was expressed as 6-acetyl glucose [6]. 

Due to the intense signal of water, ^1^H-NMR spectra were acquired using a water signal suppression pulse sequence. Considering that the pre-saturation might affect the correct quantification of signals in proximity to the frequency of the water signal (2220 Hz, 3.7 ppm), the recovery of each compound was evaluated by spiking one TBVM sample with standard compounds (Table 1). Not surprisingly, the lowest percentages of recovery were achieved by the signals of α- and β-fructofuranose, followed by β-glucopyranose and β-fructopyranose. Indeed, these peaks were the nearest to the water resonance. Notwithstanding, these signals were affected by less than 10%, demonstrating that the power level for pre-saturation did not significantly affect the surrounding peaks. Furthermore, the deconvolution process could potentially impact the accuracy of the quantification results, as it relies on an algorithm. From the results achieved from the recovery study, the effect of deconvolution could not be completely inferred from our data. In fact, fructose signals were underestimated, whereas acetoin and malic and succinic acids were overestimated. The recovery rates for all other compounds were near 100%.

The results from the quantitative analysis in NMR revealed great variability in the concentration of the main chemical constituents of vinegar samples belonging to the same group (Table 2), which was in accordance with the literature [6].

As explained above, especially for TBVM, the production process is extremely complex and influenced by the habits of the small producers even though the regulation is respected. The composition of grapes used to prepare must and wine vinegar (only for BVM) might vary extremely and influence the growth rate of microorganisms involved in fermentation, leading to a variable content of metabolites [1]. Additionally, the type and thickness of the wooden cask were demonstrated to influence the aging process [32]. Moreover, the amount of old vinegar that has been withdrawn from barrel No.5 and substituted with vinegar from barrel No.4, as well as the volume of barrels used, are crucial factors in determining the true age of TBVM [33]. This is because, as per the TBVM production process, every barrel contains a blend of various vinegar types with varying ages [1,33]. 

The SSC expressed as °Brix agreed with previous findings [24]. Overall, the concentration of the most concentrated target compounds agreed with the results obtained by other authors through both NMR and chromatographic techniques. Ethanol and the amino acids leucine and valine were identified and quantified in only a few BVM samples (<1 mg/100 g). For this reason, these results were not reported and considered for the following statistical analysis. Regarding TBVM, huge differences between old and extra-old vinegar samples in the contents of the most concentrated compounds, namely organic acids and sugars, were not detected. This evidence could be due to the fact that the real ages of the samples were unknown due to the different cask sets and procedures employed by local producers [33]. Consequently, samples belonging to the same group can vary extremely in composition, as explained above. The amounts of malic, tartaric, and acetic acids were comparable to those obtained by Sanarico and co-workers, whereas the content of sugars was slightly higher [5]. The concentration of lactic acid was also slightly higher than that reported in the literature by Cocchi et al., whereas the content of succinic acid was lower [8]. Citric acid and gluconic acids, which are organic acids present in grapes, were not detected, which was probably due to their low concentrations or overlapping with other signals in the ^1^H-NMR spectrum. 

Regarding BVM, the differences between aged and not-aged samples were more marked and mainly related to the contents of glucose and fructose. The quantitative results for not-aged BVM agreed with the results present in the literature [6]. On the contrary, scant data were previously reported for aged BVM [29]. However, a direct comparison cannot be performed due to the uncertainty of vinegar aging and production in terms of starting materials. 

**Figure 2:**
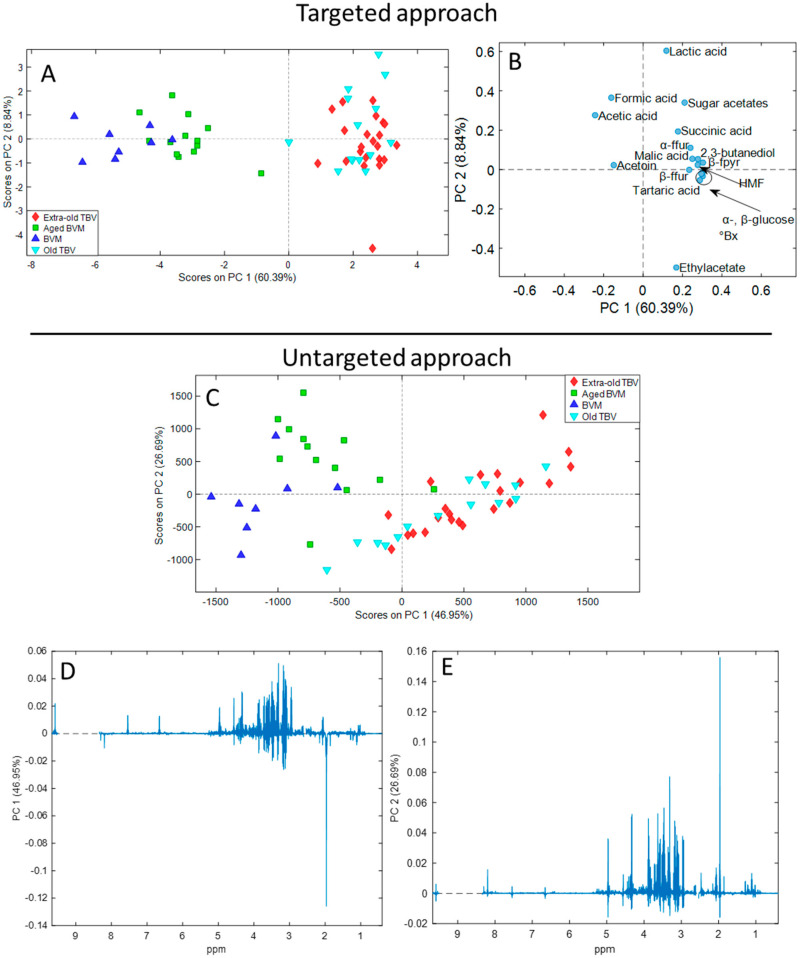
Score (**A**,**C**) and loading (**B**,**D**,**E**) plots of the principal component analyses performed on targeted (quantitative results from qNMR analysis) and untargeted (spectral points of NMR spectra) datasets.

These quantitative data were then employed for the generation of the targeted dataset for the unsupervised multivariate PCA. In parallel, PCA was performed on the untargeted dataset created by exporting fingerprinting profiles in NMR spectra as spectral points. The descriptive PCA is based on the extraction of new artificial variables (PCs) which carry the most important information present in the dataset. The results of PCA are summarized in the score (Figure 2A,C) and loading plots (Figure 2B,D,E). The score plots display the disposition of vinegar samples in the cartesian plane described by the extracted PCs, while the loading plots show the most important variables for each PC that allowed the separation of the samples in the space. The targeted PCA extracted three PCs explaining 75.61% of the total variance in the dataset, with an RMSECV equal to 0.737. The untargeted PCA extracted three PCs explaining 79.86% of the total variance, with an RMSECV equal to 3.382. The RMSECV value was acceptable for both PCA models, demonstrating their strength. The third PC of both two analyses was not reported since did not introduce any amelioration for the clustering of samples belonging to the same kind of vinegar. 

Overall, BVM and TVBM were clearly clustered in the score plots of both models, except for one aged-BVM sample which was collocated near the TVBM samples in the untargeted approach. The different kinds of vinegar were separated depending on the aging along the PC1 in the targeted model. The variables (Figure 2B) that most influenced sample projection in the space depending on the age were the higher concentrations of glucose, fructose, sugar acetates, 5-HMF, 2,3-butanediol, ethyl acetate, and succinic, lactic, malic, and tartaric acids. On the opposite, “young” BVM were placed on negative values of the PC1 for the greater contents of acetoin and acetic and formic acids. 

Regarding the untargeted model, the separation of samples depending on age was induced by both PCs. Specifically, the greatest variance was observed by PC1 between BVM and TBVM, while minor differences were described by PC2 between aged and not-aged BVM. By in-depth examination of the PC1 loading plot (Figure 2D), it was evident that the clustering of TBVM and BVM was induced by the same compounds above described. Indeed, TBVM samples were positively projected on PC1 mainly due to more intense signals of 2,3-butanediol, sugar acetates, anomeric protons of glucose, and 5-HMF at 0.98/0.93, 2.06, 4.32/4.96, and 4.56 (and 6.66, 7.54, and 9.58) ppm, respectively. Moreover, the signals of fructose tautomers (3.89, 3.63, and 3.33 ppm) were also identified at positive values. On the contrary, BVM samples were negatively projected due to more intense resonances of ethanol, acetic acid, acetoin, and formic acid at 1.11, 1,98, 1.22/2.15, and 8.20 ppm, respectively. Concerning the PC2 loading plot (Figure 2E), the signal of the acetic acid was the most important variable, followed by glucose, fructose, 5-HMF, and succinic and malic acids peaks. Certainly, for the almost complete separation of aged and not-aged BVM, the acetic acid resonance had a lower importance, since the organic acid was slightly more concentrated in young BVM. 

The results achieved through both approaches were in line with previous outcomes. Indeed, the influence of variables in the disposition of samples in the space agreed with previous studies on the chemical composition of vinegar during aging. The contents of sugars and grape organic acids (e.g., malic and tartaric acids) increase during the natural concentration process in barrels, along with those produced by the fermentation process (e.g., succinic and lactic acids), explaining the importance of such variables. The importance and increment of these compounds during aging were strictly connected to the high weight of the variable °Bx on PC1 in the targeted model, as expected. Formic acid is also produced during fermentation; however, its concentration was higher in BVM than in TBVM due to its high volatility and consequent loss during vinegar maturation. Additionally, the importance of 5-HMF, sugar acetates, and 2,3-butanediol for the aging separation once again demonstrated their importance for the monitoring of vinegar maturation [3,11,12,22,24,29]. Acetic acid and acetoin played a central role in clustering BVM on negative values of PC1 in both models accordingly to the results of other authors [24,29]. The low concentrations of these two compounds in TBVM compared to BVM are related to the different production process of the two kinds of vinegar. The unsupervised hierarchical cluster analysis was also carried out on both targeted and untargeted datasets providing a similar classification of the samples (Supplementary Materials).

**Figure 3:**
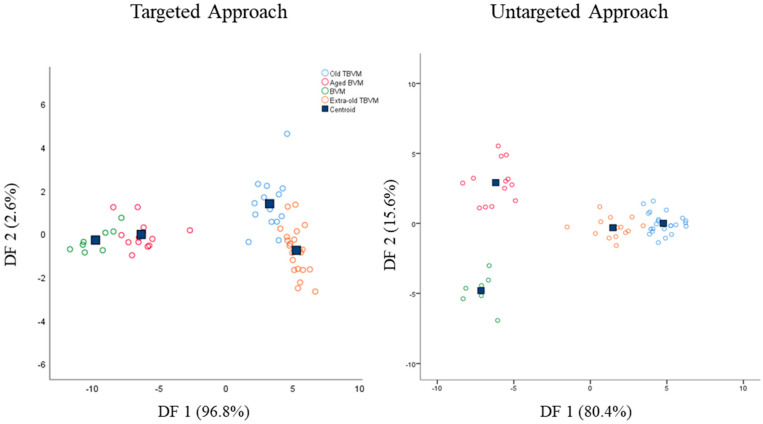
Score plots of linear discriminant analyses performed on targeted and untargeted datasets for the discrimination of different kinds of vinegar from Modena province.

Starting from the promising results achieved through the unsupervised PCA, classification models based on partial least squared discriminant analysis (PLS-DA) or linear discriminant analysis (LDA) were built and tested on internal samples (cross-validation). The PLS-DA models displayed unsatisfactory results on both the targeted and untargeted datasets (reduced and whole), with sensitivity and specificity values lower than 60% for certain groups in cross-validation (Table S1). In any case, models based on LDA showed good sensitivity and specificity values both in calibration and cross-validation (Table 3) with an almost complete clustering of samples belonging to the same class in score plots displayed in Figure 3.

This result was not surprising since LDA can be considered a more powerful tool than PLS-DA for classifying samples. Indeed, LDA achieves the highest discrimination by maximizing the ratio of the within-class and between-class distance [34]. Thus, variables are selected based on their variance within groups. Conversely, the PLS algorithm aims to capture most of the information in the variable matrix (X) useful to predict the class matrix (Y) [35]. Thus, PLS-DA shows high predictive performances for homogeneous and well-separated classes [36].

Overall, sensitivity was higher than 87.5%, while specificity was greater than 85.7%. Sensitivity is defined as the capability of the model to correctly classify samples belonging to the class; besides, specificity expresses the capability of the model to reject samples not belonging to the class. The samples correctly classified in the targeted approach were 91.2% and 89.5% in calibration and cross-validation, respectively, whereas in the untargeted approach were 98.2% and 94.7%. Thus, both models showed excellent classification capabilities. Due to their close chemical composition, the unclassified samples were mainly represented by old and extra-old TBVM. Additionally, the targeted LDA model did not discriminate one sample of aged BVM from the not-aged BVM group. For the latter reason, the sensitivity and specificity percentages of the targeted LDA model were slightly lower than the untargeted model. The greater strength of the untargeted LDA model was also noticed from the score plots reported in Figure 3, where BVM and aged BVM were clearly clustered and separated into two different groups. Both targeted and untargeted models extracted two discriminant functions (DF) explaining 99.4% and 96% of the total variance, respectively. 

In targeted LDA, the DF1 was mainly influenced in positive by °Bx, 5-HMF, 2,3-butanediol, glucose, and fructose concentrations, whereas DF2 by glucose and fructose contents in positive and 5-HMF in negative. 

Concerning the untargeted approach, the results achieved by the LDA model were strictly in agreement with previous reports where LDA or PLS-DA models were employed [23,24]. Furthermore, in this case, 5-HMF (4.56 ppm), glucose, and fructose signals (4.32, 4.96 ppm, and several between 2.93 and 3.89 ppm) had a key role in the discrimination of BVM and TBVM on both DF1 and DF2. Moreover, unknown peaks between 4.37 and 4.50 ppm and at 4.93 ppm were also employed by the discriminant functions for sample clustering. These latter signals might be the reason why the untargeted approach showed higher accuracy in sample classification. 

The results achieved through LDA models agreed with the previously discussed data obtained through PCA. Additionally, the prominent importance of 5-HMF and sugars as vinegar class predictors were strongly in accordance with the aging process above described. Models based on the PLS algorithm might have failed due to the similar chemical composition of vinegar samples used in the present study. By increasing the spectral library of vinegar samples, the accuracy of PLS-DA models might increase. 

## 4. Conclusions

The authentication and quality control of TBVM and BVM is a difficult task due to the complexity and extremely high variability of these valuable foods. In the present work, ^1^H-NMR was demonstrated to be a solid analytical tool for quantifying the most important and characteristic compounds of TBVM and BVM. Indeed, the quantitative results obtained through only one analysis were in line with those present in the literature obtained with conventional and well-known separative methods, consolidated by decades. Additionally, even though the results are only preliminary due to the limited number of samples, authentication models demonstrated the great potentiality of NMR spectroscopy coupled with chemometrics for authenticating TBVM and BVM. The targeted and the untargeted approaches efficiently provide an almost complete sample classification. The results showed that 5-HMF and sugars are the most important compounds for discriminating the types of TBVM and BVM. The untargeted models demonstrated to be the best strategy for providing rapid results for the authentication of these valuable Italian products. Indeed, the untargeted approach is certainly faster and easier based on the fingerprinting of samples. Besides, the targeted approach is time consuming since a preliminary extensive study of the best qNMR conditions is required. Moreover, important discriminant information related to unrecognized signals is lost. This preliminary study based on the application of LDA on ^1^H-NMR results laid the foundations for the setting up of a rapid tool for the quality control of vinegar that could be employed by Italian producers. Certainly, the spectral library for robust chemometric models has to be increased for hindering the extremely high variability of TBVM and BVM.

## Figures and Tables

**Table 1:** Signals used for the quantification of principal compounds in BVMs and TBVs and recovery ratios.

	Chemical Shift (ppm)	Proton	Multiplicity	Recovery %
Acetic acid	1.96	C2H3	s	98
Acetoin	2.15 1.22	C2H3 * C4H3 *	s d	104
2,3-butanediol	0.98 0.93	C1H3 C4H3	d d	105
Ethanol	1.11	C2H3	t	96
Ethyl acetate	1.18	CH3	t	99
Formic acid	8.20	HCOOH	s	101
Fructose	3.31 3.63 3.89	C1H2 β-fpyr * C1H α-ffur * C1H β -ffur *	s s s	94 92 93
Glucose	4.96 4.32	C1H α -gpyr C1H β -gpyr	d	98
			d	94
5-HMF	4.56	C6H2	s	99
Lactic acid	1.28	C3H3	t	103
Leucine	0.76	C5H3/C5′H3	d	103
Malic acid	2.45–2.62	CH2 *	dd	105
Succinic acid	2.47	C2H2/C3H2 *	s	104
Sugars acetates	2.04–2.10	C3H-CO-R	Overlapped singlets	105
Tartaric acid	4.36	C2H/C3H	s	98
Valine	0.94	C4H3/C4′H3	d	103

* signal deconvolution was employed before the integration. ffur, fructofuranose; fpyr, fructopyranose; gpyr, glucopyranose. Multiplicity: s, singlet; d, doublet; dd, doublet of doublets t, triplet.

**Table 2:** Quantitative results of target compounds in old and extra-old TBVM and BVM and aged BVM. Results were expressed as mean, standard deviation, and interval range.

		Old TBVM			Extra-Old TBVM			BVM			Aged BVM		
		Mean ± SD	Range	*	Mean ± SD	Range	*	Mean ± SD	Range	*	Mean ± SD	Range	*
Acetic acid (g/100 g)		2.25 ± 0.85	1.05–3.79	a	2.19 ± 0.83	0.96–3.71	a	5.98 ± 1.65	3.15–8.29	c	4.26 ± 1.46	2.51–6.56	b
Acetoin (g/100 g)		0.013 ± 0.006	0.005–0.025	a	0.016 ± 0.008	0.006–0.033	c	0.03 ± 0.015	0.006–0.048	b	0.023 ± 0.025	0.006–0.098	ab
2,3-butanediol (g/100 g)		0.34 ± 0.09	0.19–0.51	a	0.33 ± 0.08	0.17–0.49	b	0.052 ± 0.007	0.042–0.06	a	0.059 ± 0.011	0.039–0.077	a
Ethanol (g/100 g)		-	-		-	-		0.029 ± 0.04	ND–0.098	a	0.04 ± 0.066	ND–0.181	a
Ethyl acetate (g/100 g)		0.019 ± 0.009	ND–0.03	a	0.024 ± 0.008	0.014–0.045	a	-	-		-	-	
Formic acid (g/100 g)		0.13 ± 0.03	0.09–0.19	a	0.12 ± 0.03	0.07–0.19	a	0.17 ± 0.06	0.1–0.25	b	0.18 ± 0.06	0.09–0.32	b
Fructose (g/100 g)	β-fpyr α-ffur β-ffur Total	15.02 ± 0.87 11.65 ± 2.85 9.85 ± 2.38 36.52 ± 3.66	13.37–16.38 9.61–18.70 5.24–12.46 29.54–41.31	c b c c	14.60 ± 1.81 9.23 ± 2.25 9.34 ± 2.19 33.17 ± 4.41	10.05–17.21 6–13.64 5.30–13.84 24.83–39.87	c b c c	7.52 ± 2.71 3.71 ± 1.72 4.18 ± 1.51 15.42 ± 5.48	4.32–11.72 2.11–6.99 2.56–6.36 9.02–23.13	a a a a	10.27 ± 2.051 6.26 ± 1.68 7.22 ± 1.94 24.08 ± 4.84	8.34–14.44 5.41–9.53 5.25–11.06 17.28–31.92	b a b b
Glucose (g/100 g)	α-gpyr β-gpyr Total	14.10 ± 1.79 22.78 ± 2.54 36.88 ± 4.27	11.55–13.91 20.44–26.53 31.45–44.21	d c c	13.98 ± 1.52 22.52 ± 3.48 36.50 ± 4.27	10.82–16.13 10.02–25.51 30.90–40.92	c c c	6.48 ± 2.24 10.07 ± 3.37 16.56 ± 5.61	3.61–10.02 5.63–15.08 9.24–25.11	a a a	8.59 ± 1.24 14.19 ± 2.03 22.78 ± 3.23	6.89–10.98 11.79–17.78 19.8–29.76	b b b
5-HMF (g/100 g)		0.88 ± 0.16	0.57–1.15	c	1.24 ± 0.24	0.66–1.79	c	0.104 ± 0.024	0.06–0.14	a	0.286 ± 0.125	0.12–0.48	a
Lactic acid (g/100 g)		0.21 ± 0.08	0.12–0.38	c	0.19 ± 0.04	0.1–0.3	ab	0.13 ± 0.04	0.08–0.19	a	0.157 ± 0.045	0.097–0.233	ab
Malic acid (g/100 g)		1.65 ± 0.35	1.16–2.29	b	1.74 ± 0.53	0.58–2.84	b	0.42 ± 0.17	0.13–0.69	a	0.71 ± 0.22	0.39–1.11	a
Succinic acid (g/100 g)		0.14 ± 0.06	0.07–0.3	c	0.13 ± 0.05	0.05–0.25	bc	0.08 ± 0.02	0.06–0.13	ab	0.08 ± 0.017	0.057–0.124	a
Sugar acetates (g/100 g)		2.21 ± 0.88	1.15–4.18	c	2.52 ± 1.02	0.35–4.42	b	0.77 ± 0.21	0.54–1.18	a	1.1 ± 0.27	0.67–1.53	a
Tartaric acid (g/100 g)		0.56 ± 0.09	0.41–0.72	c	0.56 ± 0.1	0.42–0.75	c	0.11 ± 0.05	0.02–0.19	a	0.26 ± 0.13	0.17–0.63	b
°Brix		65.62 ± 2.87	60.5–70	c	71.93 ± 1.04	70–74	d	22.51 ± 5.19	15.25–32	a	38.71 ± 5.81	28.5–53.5	b

5-HMF, 5-hydroxymethylfurfural; ffur, fructofuranose; fpyr, fructopyranose; gpyr, glucopyranose. * Values in the same row with different lower-case letters are significantly different at *p* < 0.05.

**Table 3:** Sensitivity and specificity results of classification of vinegar samples from Modena province of linear discriminant analysis models for the targeted and the untargeted approaches. Results are expressed as percentages.

		Old TBVM	Extra-Old TBVM	BVM	Aged BVM
Targeted approach	Sensitivity (CAL)	92.9	90.9	87.5	92.3
	Sensitivity (CV)	85.7	90.9	87.5	92.3
	Specificity (CAL)	90.9	92.9	92.3	87.5
	Specificity (CV)	90.9	85.7	92.3	87.5
Untargeted approach	Sensitivity (CAL)	92.9	86.4	87.5	92.3
	Sensitivity (CV)	92.9	86.4	87.5	92.3
	Specificity (CAL)	86.4	92.9	92.3	87.5
	Specificity (CV)	86.4	92.9	92.3	87.5

## Data Availability

The data presented in this study are available on request from the corresponding author.

## Supplementary Materials

The following supporting information can be downloaded at: https://www.mdpi.com/article/10.3390/foods12071467/s1, Figure S1: Dendrogram from the Hierarchical Clustering Analysis performed on the targeted dataset; Figure S2: Dendrogram from the Hierarchical Clustering Analysis performed on the untargeted dataset. Table S1: Sensitivity and specificity results of classification of vinegar samples from Modena province of partial least squared discriminant analysis models for the targeted and the untargeted approaches. Results are expressed as percentages.

## References

[1] Giudici P., Gullo M., Solieri L., Solieri L., Giudici P. (2009). Traditional Balsamic Vinegar. Vinegars of the World.

[2] Tagliazucchi D., Verzelloni E., Conte A. (2010). Contribution of Melanoidins to the Antioxidant Activity of Traditional Balsamic Vinegar During Aging. J. Food Biochem..

[3] Consonni R., Gatti A. (2004). ^1^H NMR Studies on Italian Balsamic and Traditional Balsamic Vinegars. J. Agric. Food Chem..

[4] Cocchi M., Durante C., Grandi M., Lambertini P., Manzini D., Marchetti A. (2006). Simultaneous determination of sugars and organic acids in aged vinegars and chemometric data analysis. Talanta.

[5] Sanarico D., Motta S., Bertolini L., Antonelli A. (2003). HPLC Determination of Organic Acids in Traditional Balsamic Vinegar of Reggio Emilia. J. Liq. Chromatogr. Relat. Technol..

[6] Lalou S., Hatzidimitriou E., Papadopoulou M., Kontogianni V.G., Tsiafoulis C.G., Gerothanassis I.P., Tsimidou M.Z. (2015). Beyond traditional balsamic vinegar: Compositional and sensorial characteristics of industrial balsamic vinegars and regulatory requirements. J. Food Compos. Anal..

[7] Antonelli A., Chinnici F., Masino F. (2004). Heat-induced chemical modification of grape must as related to its concentration during the production of traditional balsamic vinegar: A preliminary approach. Food Chem..

[8] Cocchi M., Lambertini P., Manzini D., Marchetti A., Ulrici A. (2002). Determination of Carboxylic Acids in Vinegars and in Aceto Balsamico Tradizionale di Modena by HPLC and GC Methods. J. Agric. Food Chem..

[9] Plessi M., Bertelli D., Miglietta F. (2006). Extraction and identification by GC-MS of phenolic acids in traditional balsamic vinegar from Modena. J. Food Compos. Anal..

[10] Verzelloni E., Tagliazucchi D., Conte A. (2007). Relationship between the antioxidant properties and the phenolic and flavonoid content in traditional balsamic vinegar. Food Chem..

[11] Consonni R., Cagliani L.R., Rinaldini S., Incerti A. (2008). Analytical method for authentication of Traditional Balsamic Vinegar of Modena. Talanta.

[12] Cirlini M., Caligiani A., Palla L., Palla G. (2011). HS-SPME/GC–MS and chemometrics for the classification of Balsamic Vinegars of Modena of different maturation and ageing. Food Chem..

[13] Guerrero E.D., Chinnici F., Natali N., Marín R.N., Riponi C. (2008). Solid-phase extraction method for determination of volatile compounds in traditional balsamic vinegar. J. Sep. Sci..

[14] Theobald A., Müller A., Anklam E. (1998). Determination of 5-Hydroxymethylfurfural in Vinegar Samples by HPLC. J. Agric. Food Chem..

[15] Bertelli D., Papotti G., Bortolotti L., Marcazzan G.L., Plessi M. (2011). 1H-NMR Simultaneous Identification of Health-Relevant Compounds in Propolis Extracts. Phytochem. Anal..

[16] Bertelli D., Lolli M., Papotti G., Bortolotti L., Serra G., Plessi M. (2010). Detection of Honey Adulteration by Sugar Syrups Using One-Dimensional and Two-Dimensional High-Resolution Nuclear Magnetic Resonance. J. Agric. Food Chem..

[17] Truzzi E., Marchetti L., Fratagnoli A., Rossi M.C., Bertelli D. (2023). Novel application of 1H NMR spectroscopy coupled with chemometrics for the authentication of dark chocolate. Food Chem..

[18] Sobolev A.P., Ingallina C., Spano M., Di Matteo G., Mannina L. (2022). NMR-Based Approaches in the Study of Foods. Molecules.

[19] Ingallina C., Cerreto A., Mannina L., Circi S., Vista S., Capitani D., Spano M., Sobolev A.P., Marini F. (2019). Extra-Virgin Olive Oils from Nine Italian Regions: An 1H NMR-Chemometric Characterization. Metabolites.

[20] Papotti G., Bertelli D., Graziosi R., Silvestri M., Bertacchini L., Durante C., Plessi M. (2012). Application of One- and Two-Dimensional NMR Spectroscopy for the Characterization of Protected Designation of Origin Lambrusco Wines of Modena. J. Agric. Food Chem..

[21] Consonni R., Cagliani L.R. (2019). The potentiality of NMR-based metabolomics in food science and food authentication assessment. Magn. Reson. Chem..

[22] Consonni R., Cagliani L. (2007). NMR relaxation data for quality characterization of Balsamic vinegar of Modena. Talanta.

[23] Consonni R., Cagliani L., Benevelli F., Spraul M., Humpfer E., Stocchero M. (2008). NMR and Chemometric methods: A powerful combination for characterization of Balsamic and Traditional Balsamic Vinegar of Modena. Anal. Chim. Acta.

[24] Papotti G., Bertelli D., Graziosi R., Maietti A., Tedeschi P., Marchetti A., Plessi M. (2015). Traditional balsamic vinegar and balsamic vinegar of Modena analyzed by nuclear magnetic resonance spectroscopy coupled with multivariate data analysis. LWT.

[25] Bertelli D., Maietti A., Papotti G., Tedeschi P., Bonetti G., Graziosi R., Brandolini V., Plessi M. (2014). Antioxidant Activity, Phenolic Compounds, and NMR Characterization of Balsamic and Traditional Balsamic Vinegar of Modena. Food Anal. Methods.

[26] Graziosi R., Bertelli D., Marchetti L., Papotti G., Rossi M.C., Plessi M. (2017). Novel 2D-NMR Approach for the Classification of Balsamic Vinegars of Modena. J. Agric. Food Chem..

[27] Truzzi E., Marchetti L., Benvenuti S., Righi V., Rossi M.C., Gallo V., Bertelli D. (2021). A Novel qNMR Application for the Quantification of Vegetable Oils Used as Adulterants in Essential Oils. Molecules.

[28] Association of Official Analytical Chemistry (1932). AOAC Official Method 932.12 Solids (Soluble) in Fruits and Fruit Products. https://www.scribd.com/document/404250975/AOAC-Official-Method-932-12-Solids-Soluble-in-Fruits-and-Fruit-Products-pdf.

[29] Caligiani A., Acquotti D., Palla G., Bocchi V. (2007). Identification and quantification of the main organic components of vinegars by high resolution 1H NMR spectroscopy. Anal. Chim. Acta.

[30] Barclay T., Ginic-Markovic M., Johnston M.R., Cooper P., Petrovsky N. (2012). Observation of the keto tautomer of d-fructose in D2O using 1H NMR spectroscopy. Carbohydr. Res..

[31] Zhu Y., Zajicek J., Serianni A.S. (2001). Acyclic Forms of [1-^13^C]Aldohexoses in Aqueous Solution: Quantitation by ^13^C NMR and Deuterium Isotope Effects on Tautomeric Equilibria. J. Org. Chem..

[32] Torija M.-J., Mateo E., Vegas C.-A., Jara C., González A., Poblet M., Reguant C., Guillamon J.-M., Mas A. (2009). Effect of Wood Type and Thickness on Acetification Kinetics in Traditional Vinegar Production. Int. J. Wine Res..

[33] Giudici P., Rinaldi G. (2007). A theoretical model to predict the age of traditional balsamic vinegar. J. Food Eng..

[34] Todorov V. (2007). Robust selection of variables in linear discriminant analysis. Stat. Methods Appl..

[35] Garthwaite P.H. (1994). An Interpretation of Partial Least Squares. J. Am. Stat. Assoc..

[36] Fordellone M., Bellincontro A., Mencarelli F. (2020). Partial least squares discriminant analysis: A dimensionality reduction method to classify hyperspectral data. Stat. Appl.-Ital. J. Appl. Stat..

