# Supplementary material for: Multivariate Statistical Models for the Authentication of Traditional Balsamic Vinegar of Modena and Balsamic Vinegar of Modena on 1H-NMR Data: Comparison of Targeted and Untargeted Approaches
Source: Foods. 2023 Mar 29;12(7):1467. doi: 10.3390/foods12071467 (PMC10093814; doi:10.3390/foods12071467)
Supplement: Supplementary file 1 [file foods-12-01467-s001.zip › foods-2296486-supplementary.pdf]

## *Hierarchical cluster analysis*

Hierarchical cluster analysis (HCA) was performed on both targeted and untargeted datasets on PLS_Toolbox for MATLAB® (version 8.9.2, Mathworks Inc., Natick, MA, USA). Quantitative data were preprocessed by autoscaling, while spectral data were pretreated by means of baseline correction followed by Pareto-scaling and mean-centering. The k-means algorithm was used for the untargeted HCA on both datasets.

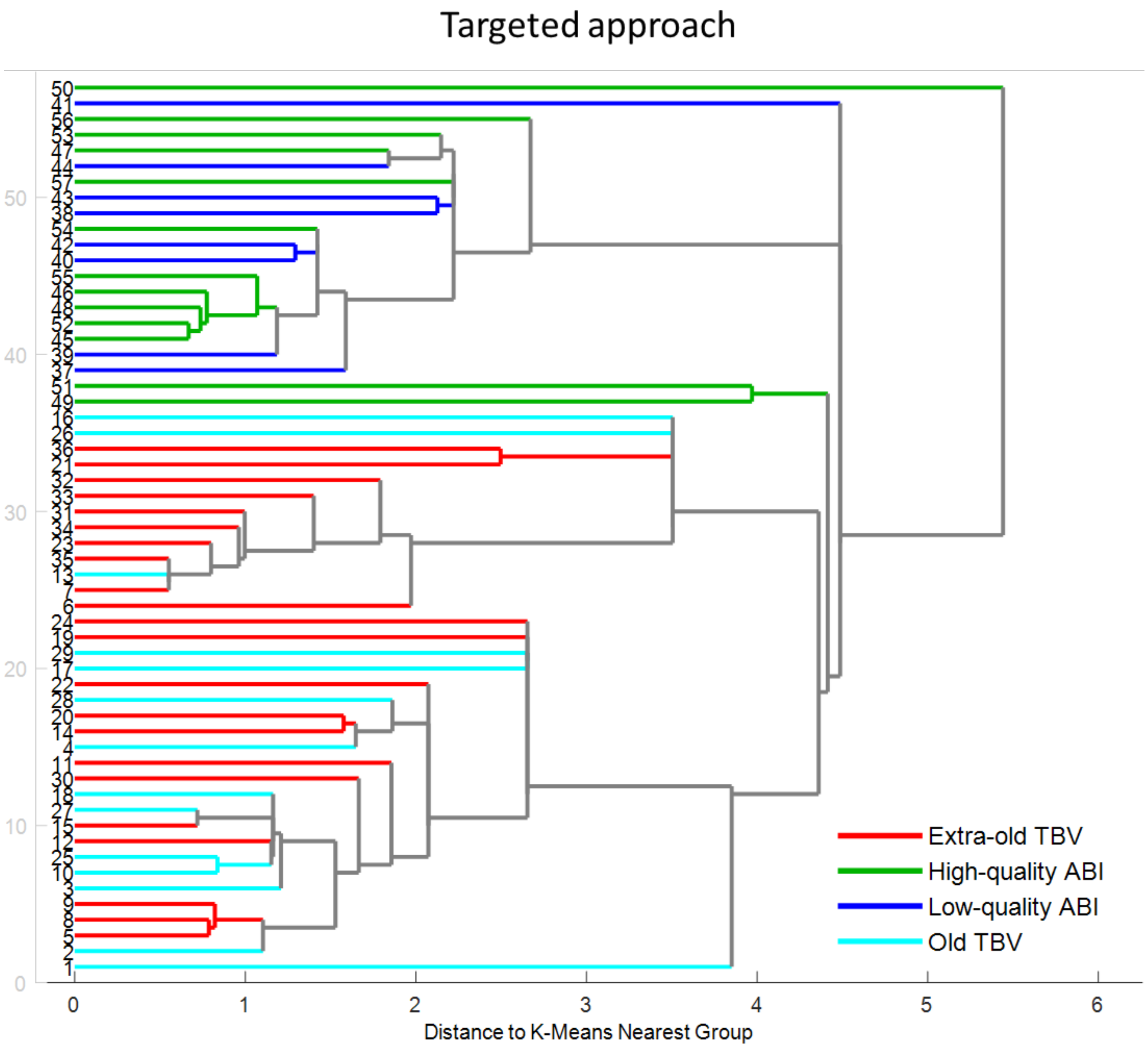


**Figure S1.** Dendrogram from the Hierarchical Clustering Analysis performed on the targeted dataset.

Untargeted approach

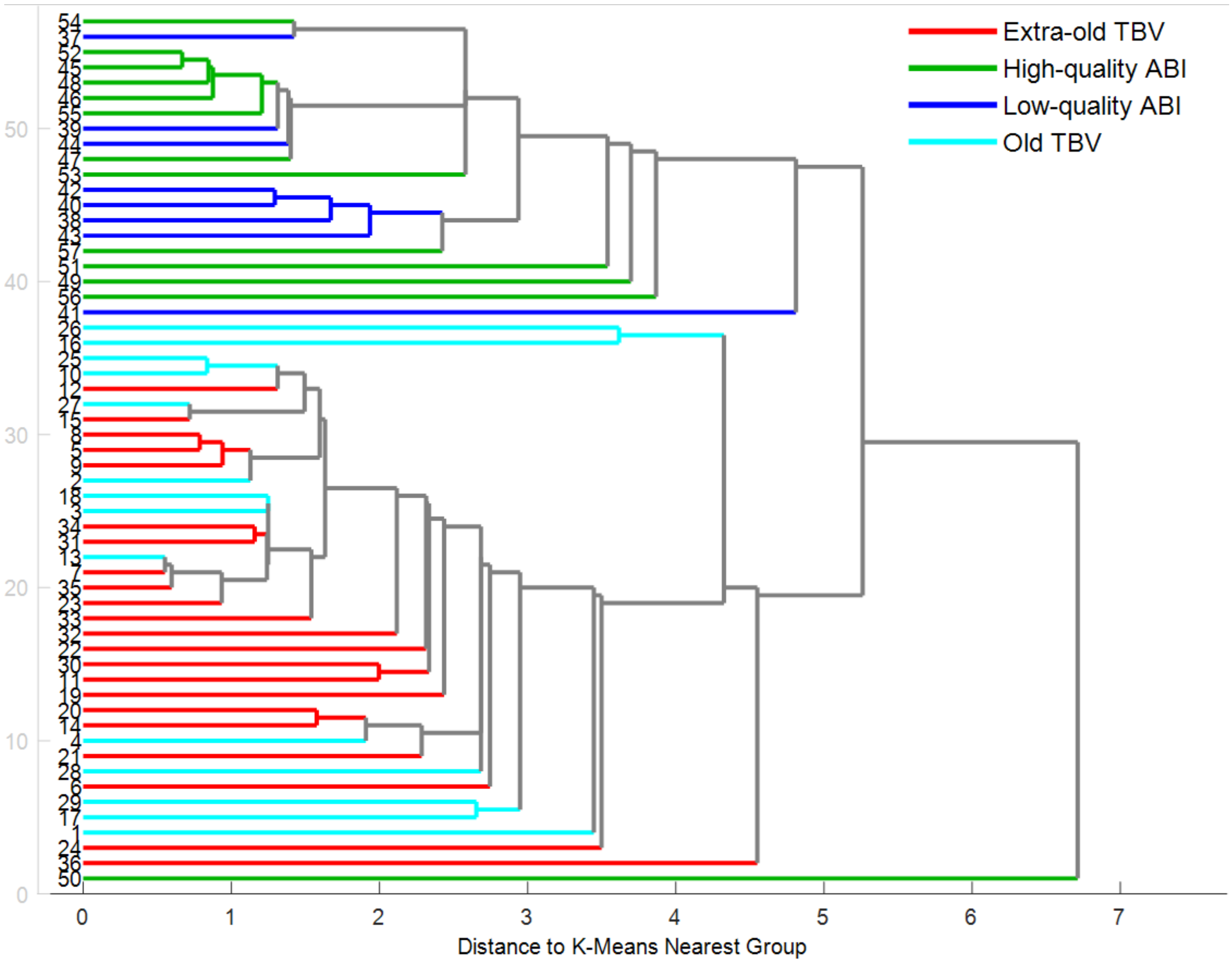


**Figure S2.** Dendrogram from the Hierarchical Clustering Analysis performed on the untargeted dataset.

### *Partial least squared discriminant analysis (PLS-DA)*

Partial least squared discriminant analysis was performed on PLS_Toolbox for MATLAB® (version 8.9.2, Mathworks Inc., Natick, MA, USA) on the targeted and untargeted datasets. The cross-validation for all the statistical models was performed by using the leave-one-out method. The number of latent variables (LVs) was selected according to the smallest root mean squared error in calibration (RMSEC) and cross-validation (RMSECV).

**Table S1**. Sensitivity and specificity results of classification of vinegar samples from Modena province of partial least squared discriminant analysis models for the targeted and the untargeted approaches. Results are expressed as percentages.

| | | Old TBVM | Extra-Old TBVM | BVM | Aged BVM |
|---|---|---|---|---|---|
| **Targeted approach** | Sensitivity (CAL) | 90.9 | 92.3 | 87.5 | 64.3 |
| | Sensitivity (CV) | 86.4 | 84.6 | 87.5 | 50 |
| | Specificity (CAL) | 85.7 | 81.8 | 91.8 | 83.7 |
| | Specificity (CV) | 74.3 | 84.1 | 85.7 | 76.7 |
| **Untargeted approach** | Sensitivity (CAL) | 82.6 | 69.2 | 92.3 | 88.9 |
| | Sensitivity (CV) | 78.3 | 61.5 | 92.3 | 77.8 |
| | Specificity (CAL) | 88.6 | 88.9 | 91.1 | 95.9 |
| | Specificity (CV) | 85.7 | 86.7 | 88.9 | 93.9 |
